# Co-exposure to microplastics and bisphenol A increases viral susceptibility in largemouth bass (*Micropterus salmoides*) via oxidative stress

**DOI:** 10.1007/s44307-025-00085-5

**Published:** 2025-11-06

**Authors:** Jie Gao, Junzhe Zhang, Rui Zheng, Jing Jiang, Siyou Huang, Qijin Miao, Bingya Wu, Wanting Tang, Jianguo He, Junfeng Xie

**Affiliations:** State Key Laboratory of Biocontrol, Southern Marine Science and Engineering Guangdong Laboratory (Zhuhai), China-ASEAN Belt and Road Joint Laboratory On Mariculture Technology, Guangdong Provincial Key Laboratory of Aquatic Economic Animals, School of Life Sciences, Sun Yat-Sen University, Guangzhou, 510275 China

**Keywords:** *Micropterus salmoides*, MPs, BPA, Oxidative stress, NNV

## Abstract

Microplastics (MPs) and bisphenol A (BPA) frequently co-occur in freshwater ecosystems, yet their combined impacts on host–pathogen interactions remain poorly understood. Here, we exposed juvenile largemouth bass (*Micropterus salmoides*) to environmentally relevant concentrations of MPs, BPA, and their mixture. Co-exposure markedly inhibited NRF2-mediated antioxidant signaling, leading to downregulation of antioxidant enzymes (SOD1, CAT, GPx), elevated hepatic reactive oxygen species and malondialdehyde, and depletion of ATP. These redox disturbances were accompanied by mitochondrial damage, increased expression of pro-apoptotic genes (Bax, Caspase-3), and accumulation of TUNEL-positive nuclei, indicative of apoptosis. Strikingly, only co-exposed fish exhibited enhanced replication of nervous necrosis virus (NNV), a response absent under single exposures. Our findings demonstrate that MPs and BPA act synergistically to disrupt redox homeostasis and compromise antiviral defense, thereby heightening viral susceptibility in a freshwater aquaculture species. This study highlights the overlooked infection risks posed by pollutant mixtures and emphasizes the need to incorporate mixture toxicity into freshwater ecotoxicological risk assessments.

**Supplementary Information:**

The online version contains supplementary material available at 10.1007/s44307-025-00085-5.

## Introduction

Microplastics (MPs) and bisphenol A (BPA) are globally ubiquitous contaminants of increasing concern due to their widespread distribution in the environment and potential risks to aquatic ecosystems. MPs, defined as plastic particles < 5 mm, exhibit highly variable abundances in both marine and freshwater environments, with concentrations ranging from 50–187,000 particles/m^3^ (Yang et al. 2022; Duan et al. 2021; Liu et al. 2023; Li et al. 2024). Notably, even remote freshwater systems such as the Tibetan Plateau have reported MP densities of 483–967 particles/m^3^ (Jiang et al. 2019), while urban rivers, such as the Pearl River, contain concentrations reaching up to 19,860 particles/m^3^ (Yan et al. 2019), reflecting the substantial impact of anthropogenic activities on MP distribution and dispersal (Liu et al. 2023).

BPA, a hydrophobic endocrine-disrupting compound widely used in plastics and resins, is frequently co-detected with MPs owing to its strong affinity for polymer surfaces (Paolella et al. 2024; Tang et al. 2023). Although often regarded as a marine pollutant, BPA is increasingly detected in freshwater systems worldwide, with surface water concentrations of 1.7–93 ng/L globally (Liang et al. 2023), 3.1–120 ng/L in Japan, 45–213 ng/L in Korea, and 54–512 ng/L in India (Yamazaki et al. 2015). In the Pearl River Delta, sedimentary BPA levels reach 0.27–120 ng/g dry weight (Liang et al. 2023), reflecting continuous inputs from industrial and domestic sources. Its detection in aquaculture environments and edible fish tissues raises additional concerns about ecological and human health risks (Lee et al. 2015; Barboza et al. 2020).

Both MPs and BPA independently induce oxidative stress and apoptosis, impairing key physiological processes in aquatic species (Liu et al. 2020; Tao et al. 2016). Recent evidence shows additive or synergistic toxicity of MP–BPA mixtures, disrupting redox balance more severely than single exposures (Sun et al. 2021; Sun et al. 2022). However, the potential of MPs to enhance BPA bioavailability and the resulting effects on host homeostasis and disease susceptibility remain poorly understood (Tang et al. 2022; Zheng et al. 2023; Burgos-Aceves et al. 2021). Addressing whether such co-exposure impairs host immunity via redox dysregulation under environmentally relevant conditions is essential for advancing risk assessment of mixed contaminants.

Largemouth bass (*Micropterus salmoides*) is a widely cultured freshwater species in China due to its rapid growth, high temperature tolerance, and market demand. Since its introduction to Guangdong in the 1980 s, annual production has exceeded 100,000 tons, positioning it as one of the most economically important fish in Chinese inland aquaculture (Yan et al. 2025; Bai et al. 2008). However, intensive farming practices have been associated with declining disease resistance, emphasizing the urgency to elucidate how environmental pollutants may impact host immunity (Cain et al., 2022).

Nervous necrosis virus (NNV), a neurotropic pathogen with global distribution, infects over 120 marine and freshwater fish species, causing viral encephalopathy and retinopathy (Huang et al. 2024; Gao et al. 2025). NNV outbreaks result in high mortality rates and characteristic neurological symptoms, including erratic swimming, anorexia, and lethargy (Huang et al. 2024; Lama et al. 2022). Viral replication has been associated with oxidative stress in host tissues, and several viruses, including NNV, are known to exploit redox imbalance to evade immune defense (Chang et al. 2011).

The transcription factor NRF2 plays a central role in orchestrating antioxidant responses by inducing expression of superoxide dismutase (*SOD1*), catalase (*CAT*), glutathione peroxidase (*GPx1*) genes. Its inhibition, whether by pollutant exposure or viral infection, trggers redox collapse and apoptosis (Chang et al. 2011; Jung et al., 2010; Nguyen et al. 2009; Lee et al., 2018; Xu et al. 2020; Ping et al. 2024). Previous studies have suggested that pollutant-induced NRF2 inhibition and oxidative stress may impair host antiviral defenses (Lekshmi et al. 2023; Camini et al. 2017). Based on this, we hypothesize that co-exposure to MPs and BPA disrupts NRF2-mediated antioxidant defense, leading to energy depletion, redox imbalance, and increased susceptibility to NNV infection.

In this study, juvenile *M. salmoides* were exposed to environmentally relevant concentrations of MPs and BPA, individual and combined, followed by NNV challenge. Multiple biochemical and molecular endpoints were examined to evaluate oxidative stress, apoptosis, and viral load. By framing NRF2 inhibition as the molecular initiating event (MIE) within an adverse outcome pathway (AOP) framework, we aim to elucidate the mechanistic links between mixed contaminant exposure and compromised antiviral defense in a key aquaculture species.

## Materials and methods

### Animal husbandry

*M*. *salmoides*, (average length 6.2 ± 0.4 cm, weight 6.8 ± 0.7 g) were purchased from Chengyi Freshwater Hatchery (Guangzhou, Guangdong, China). Prior to exposure, fish were acclimated for 7 days in a recirculating freshwater system under controlled conditions: 25 ± 1 °C, 12 h light/12 h dark cycle, dissolved oxygen > 6.0 mg/L, and pH 7.5 ± 0.2. Fish were fed a commercial diet twice daily throughout the acclimation and exposure periods.

### Preparation of BPA and microplastic solutions

BPA, (≥ 99% purity) was purchased from Sigma-Aldrich (USA) and dissolved in absolute ethanol to prepare a 1 g/L stock solution. The stock was stored at 4 °C in the dark and diluted to working concentrations as needed. BPA solutions were renewed daily to ensure stability throughout the exposure period. Green fluorescent polyethylene microplastic particles (5 μm diameter, high-density polyethylene, Cospheric LLC, USA) were used to simulate environmentally relevant microplastic exposure. A 1 g/L stock suspension was prepared in ultrapure water and ultrasonicated (150 W, 30 min, intermittent mode) to ensure homogeneous dispersion. Working suspensions were freshly prepared and thoroughly mixed with the exposure water prior to use. In the combined exposure group, BPA and microplastic suspensions were added simultaneously. A 14-day preliminary trial was conducted prior to the formal experiment, and final exposure concentrations were determined based on the results of this trial. Pristine polyethylene MPs were used without UV-aging or environmental weathering treatment.

### Experimental design

To determine appropriate exposure concentrations for the formal experiment, juvenile *M. salmoides* were separately exposed to a series of MPs: 5, 10, 15, 20, 25 μg/L) and BPA: 50, 80, 100, 150, 200 ng/L concentrations for 14 days. Survival rates were recorded daily throughout the exposure period. Based on the observed dose-dependent mortality, 10 μg/L for MPs and 100 ng/L for BPA were selected as the final exposure concentrations used in the main experiment. Five groups were established with three replicate tanks per group (30 fish per tank): (1) blank control, (2) solvent control (0.001% ethanol), (3) MPs, (4) BPA, and (5) combined exposure (MPs + BPA). The exposure lasted for 14 days, during which half of the tank water was renewed daily and toxicant concentrations were maintained by replenishment. All fish were fed normally during the experiment. At days 0, 7, and 14, six fish per group were randomly sampled for hepatic tissue collection. Samples were used for enzyme activity assays, Quantitative real-time PCR (qRT-PCR) analysis, histological staining, and ATP measurement.

### Enzyme activity assays

Liver tissues were immediately frozen in liquid nitrogen after dissection and stored at − 80 °C until analysis. Tissue homogenates were prepared in pre-cooled physiological saline, followed by centrifugation at 12,000 rpm for 15 min at 4 °C. The supernatant was collected for enzymatic analysis. Commercial assay kits (Nanjing Jiancheng Bioengineering Institute, China) were used to determine the activity or content of SOD1, CAT, GPx, malondialdehyde (MDA), aspartate aminotransferase (AST), alanine aminotransferase (ALT), and adenosine triphosphate (ATP), according to the manufacturer’s instructions. Caspase-3 activity was measured using a colorimetric kit (Elabscience, Shanghai, China), based on the hydrolysis of the Ac-DEVD-pNA substrate and detection of pNA release at 405 nm. All assays were performed in technical triplicates, and absorbance or fluorescence was recorded using a microplate reader (BioTek H1MF, USA).

### qRT-PCR analysis

Total RNA was extracted from liver tissues using TRIzol reagent (Invitrogen, USA) according to the manufacturer’s protocol. RNA concentration and purity were assessed using a NanoDrop 2000 spectrophotometer (Thermo Scientific, USA). First-strand cDNA was synthesized from 1 μg of total RNA using the PrimeScript RT reagent kit (Takara, Japan). qRT-PCR was performed on a QuantStudio 5 Real-Time PCR System (Applied Biosystems, USA) using SYBR Green Master Mix (Servicebio, China) in a final volume of 10 *μ*L. The thermal cycling conditions included an initial denaturation at 95 °C for 30 s, followed by 40 cycles of 95 °C for 5 s and 60 °C for 30 s. Each sample was tested in technical triplicates. Gene-specific primers for *NRF2*, *Keap1*, *SOD1*, *CAT*, *GPx1*, *HSP70*, *Bax*, *Bcl*-*2*, and *Caspase-3* were designed from *M. salmoides* sequences. The primers are listed in Table S1, and amplification efficiencies were > 90%. *EF*-*1α* was selected as the internal reference gene because its expression was unaffected by MPs, BPA, or NNV treatments in this study. Relative expression levels (vs. the control group) were calculated using the 2^ − ΔΔCT method.

### Histological and ultrastructural examination

To assess histopathological and ultrastructural changes induced by co-exposure to BPA and MPs, liver tissues were collected at the end of the exposure period. Hematoxylin and eosin (HE) staining and terminal deoxynucleotidyl transferase dUTP nick-end labeling (TUNEL) assay were performed. For HE staining, liver samples were fixed in 4% paraformaldehyde at room temp erature for 24 h. Samples were dehydrated through graded ethanol, embedded in paraffin, and sectioned at 5 μm thickness. Sections were stained following standard protocols and observed under a light microscope (Leica DM3000) to evaluate tissue structure, hepatocyte morphology, and inflammatory infiltration. TUNEL staining was performed using an in situ Cell Death Detection Kit (Roche, Germany). Paraffin sections were dewaxed, rehydrated, treated with proteinase K, and incubated with TUNEL reaction mixture according to the manufacturer’s instructions. Apoptotic cells were visualized by fluorescence microscopy. Apoptotic index was calculated from five randomly selected fields per sample. For ultrastructural analysis, brain tissues from control and treated fish were fixed in 2.5% glutaraldehyde at 4 °C for 24 h, followed by post-fixation in 1% osmium tetroxide for 2 h. Samples were dehydrated in a graded ethanol series and embedded in Spurr’s resin. Ultrathin sections (~ 50 nm) were cut using an ultramicrotome, stained with uranyl acetate and lead citrate, and examined under a JEOL JEM-1400FLASH transmission electron microscope (TEM) to observe mitochondrial morphology. Representative images were acquired from three biological replicates per group.

### Reactive oxygen species (ROS) fluorescence staining

ROS in liver tissue were assessed using frozen Sects. (10 μm). Sections were treated with autofluorescence quencher and incubated with DCFH-DA staining solution at 37 °C for 30 min in the dark. After PBS washes, nuclei were counterstained with DAPI. Slides were mounted and observed under a fluorescence microscope. ROS signals were visualized in the FITC channel, and nuclei were identified in the DAPI channel.

### Molecular docking and network toxicology analysis

To assess the potential interaction between BPA and NRF2 in *M. salmoides*, molecular docking was performed using AutoDock Vina. The amino acid sequence of NRF2 (Gene ID: 119,904,119) was retrieved from the NCBI database, and its 3D structure was predicted using AlphaFold2. The 3D structure of BPA was downloaded from the PubChem database. Docking simulations were conducted on the predicted ligand-binding surface of NRF2, and binding affinity (kcal/mol) and key interacting residues were analyzed using PyMOL and Discovery Studio Visualizer.

To further explore potential molecular targets and pathways, network toxicology analysis was performed. BPA- and microplastics-related target proteins were retrieved from the GeneCards database (https://www.genecards.org/), and overlapping targets were identified using the Jvenn online tool (https://jvenn.toulouse.inrae.fr/). Protein names were converted to gene symbols via the UniProt database (https://www.uniprot.org/). The shared gene list was subjected to Gene Ontology (GO) (biological process, cellular component, and molecular function) and Kyoto Encyclopedia of Genes and Genomes (KEGG) pathway enrichment analysis using Metascape (https://www.metascape.org/). Enriched terms with *P* < 0.01 were visualized using bar plots to identify the major pathways involved in MPs and BPA co-exposure.

### NNV challenge experiment

After 14 days of exposure to MPs and/or BPA, fish were transferred to clean, contaminant-free freshwater for viral challenge. NNV infection was conducted by immersion. Fish were exposed to NNV-containing water (2 × 10^2^ TCID₅₀/mL) for 6 h. Samples were collected at 0, 3, and 7 days post-infection (dpi). At each time point, three fish per group were randomly selected. Brain tissues were harvested for viral load analysis. Total RNA was extracted, and viral RNA levels were measured by qRT-PCR. The goal was to assess whether prior pollutant exposure increased susceptibility to NNV infection.

#### Statistical analysis

Data are presented as mean ± SD (n = 6 biological replicates per group per time point). Analyses were performed in GraphPad Prism 10.1. For endpoints measured across Treatment (Control, Solvent, MPs, BPA, MPs + BPA) and Time (0, 7, 14 d), we used two-way ANOVA (factors: Treatment, Time; Treatment × Time interaction). If the interaction was significant, Tukey’s multiple comparisons were conducted within each time point; otherwise, main effects were compared with Tukey’s test. Single–time-point endpoints used one-way ANOVA with Tukey’s test. Normality and homoscedasticity (Shapiro–Wilk, Brown–Forsythe) were checked; data were transformed as needed. *P* < 0.05 was considered significant.

## Results

### Selection of environmentally relevant exposure concentrations

**Fig. 1:**
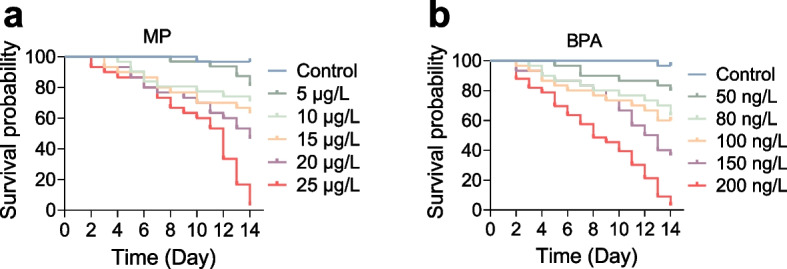
Survival of *M. salmoides* exposed MPs and BPA. Kaplan–Meier curves show concentration-dependent decreases in survival following exposure to microplastics (MPs; 5–25 μg/L) and bisphenol A (BPA; 50–200 ng/L). Sublethal concentrations (10 μg/L MPs and 100 ng/L BPA) were selected for subsequent experiments

The preliminary trial showed dose-dependent mortality (Fig. 1) response to both pollutants, with the highest death rate observed at 25 μg/L MPs and 200 ng/L BPA. In contrast, negligible mortality occurred at 10 μg/L MPs and 100 ng/L BPA, prompting these lower concentrations as the exposure benchmarks for the formal experiment.

To verify whether the selected doses facilitated MP bioaccumulation in target tissues, time-course fluorescent imaging was conducted (Fig. S1). Notably, liver morphology showed visible hypertrophy in the MPs + BPA treatment group after 14 days (Fig. S1B). Fluorescent microscopy further revealed a progressive escalation of green-labeled MPs in liver tissue (Fig. S1C), with the MPs + BPA group exhibiting significantly higher accumulation compared to MPs-alone controls.

### Antioxidant disruption induced by MPs and BPA

**Fig. 2:**
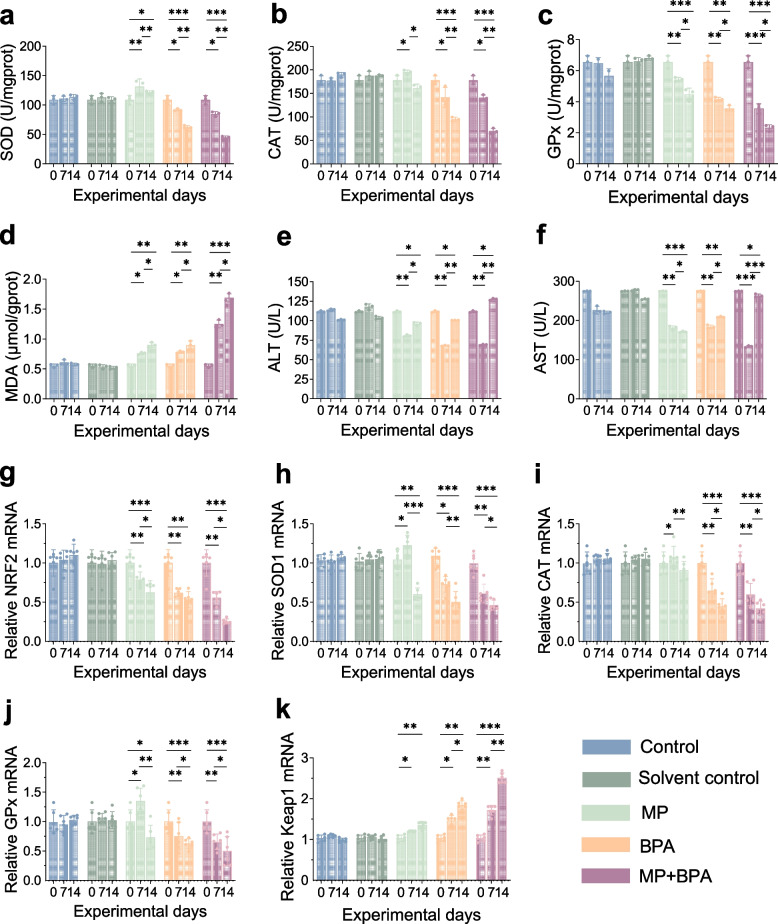
Biochemical and transcriptional responses in the liver of *M. salmoides* following MPs and BPA exposure. **a–c** Activities of antioxidant enzymes (SOD1, CAT, GPx); (d) lipid peroxidation marker (MDA); **e**, **f** liver function indicators (ALT, AST); **g–k** hepatic mRNA levels of NRF2 signaling-related genes (NRF2, SOD1, CAT, GPx1, Keap1) on days 0, 7, and 14. The MPs + BPA group showed the strongest alterations across both biochemical and gene expression endpoints. Data are presented as mean ± SD (*n* = 6); *P* < 0.05, *P* < 0.01, *P* < 0.001

Compared with the control group, exposure to MPs, BPA, or their combination markedly disrupted the hepatic antioxidant system in *M. salmoides*. Enzyme activity assays revealed significant reductions in SOD1, CAT, and GPx activities across all treatment groups, with the greatest suppression observed in the MPs + BPA group (*P* < 0.05, Fig. 2A–C). Concurrently, qRT-PCR demonstrated downregulated transcription of *SOD1*, *CAT*, *GPx1*, and *NRF2*, whereas *Keap1* expression was significantly upregulated (Fig. 2G–K). In addition, MDA content, a bioindicator of lipid peroxidation, was significantly elevated in the exposed groups, peaking in the combined MPs + BPA treatment (Fig. 2D). These results indicate that MPs and BPA, especially in combination, impair hepatic antioxidant capacity by inhibiting NRF2-mediated signaling and exacerbating oxidative stress.

### Hepatic histopathology and liver function indicators

**Fig. 3:**
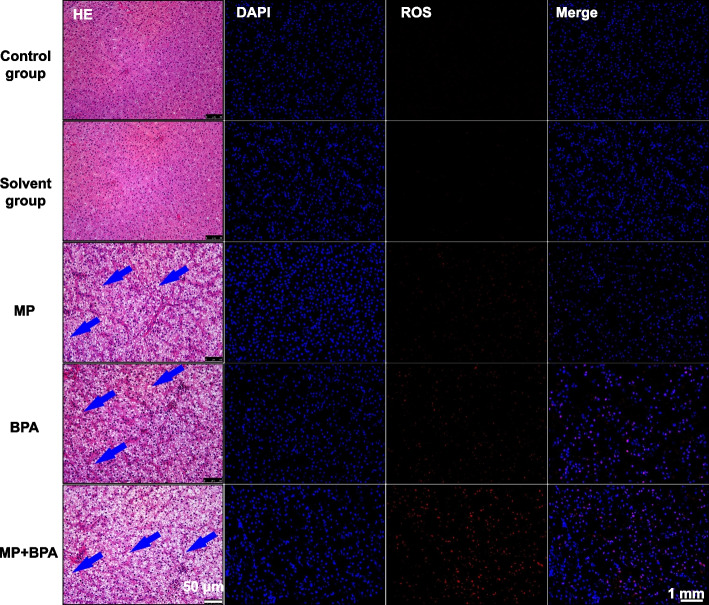
Histological alterations and ROS distribution in the liver of *M. salmoides* after exposure to MPs and BPA. Liver sections from control, solvent control, MP, BPA, and MP + BPA groups. HE staining shows vacuolization, cytoplasmic degeneration, and hepatocyte necrosis in exposed groups, with the most severe lesions observed under combined exposure (scale bar = 50 μm). Blue arrows indicate typical pathological features. DCFH-DA and DAPI staining show ROS (red) and nuclei (blue), respectively. ROS-positive signals increased markedly in the MP + BPA group compared to controls, indicating enhanced oxidative stress after combined exposure (scale bar = 1 mm)

HE staining revealed distinct histopathological alterations in the liver tissues of exposed groups. The control and solvent control livers retained normal architecture (Fig. 3A-B), whereas the MPs group showed mild vacuolization and sinusoidal dilation (Fig. 3C). The BPA group presented disorganized hepatic cords and cytoplasmic degenerative signs (Fig. 3D). The MPs + BPA group displayed the most severe lesions, including extensive vacuolization, hepatocellular necrosis, and nuclear pyknosis (Fig. 3E). Consistent with these histological damages, hepatic homogenate ALT and AST levels were significantly elevated across all exposure groups, with the highest values detected in the co-exposure group (Fig. 2E–F).

### ROS Accumulation in liver tissue

To validate oxidative stress induction, hepatic ROS distribution was examined using DCFH-DA fluorescent staining (Fig. 3). Compared to control groups, ROS-related red fluorescence were markedly intensified in the MPs and BPA groups, with the highest signal observed in the MPs + BPA group. This visual evidence was consistent with the biochemical results, including suppressed antioxidant enzyme activities and increased MDA levels, supporting the notion that co-exposure to MPs and BPA aggravates hepatic oxidative stress in *M. salmoides*.

### Stress and apoptosis signaling activation

**Fig. 4:**
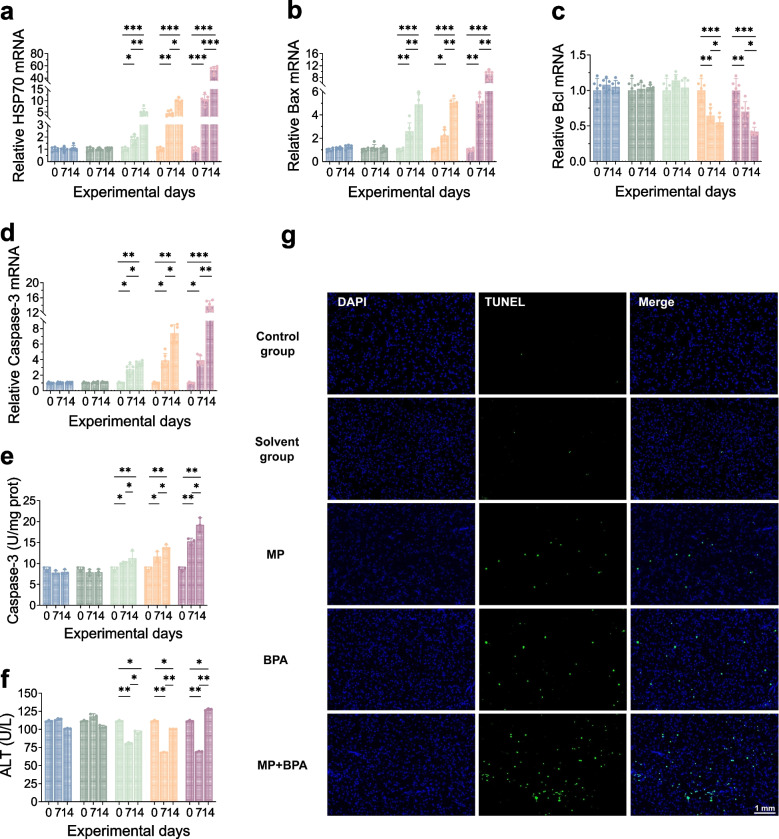
Stress and apoptosis responses in *M. salmoides* following MPs and BPA exposure.** a** HSP70 mRNA expression; **b–d** expression of Bax, Bcl-2, and Caspase-3; **e** Caspase-3 enzyme activity; **f** hepatic ATP content; **g** TUNEL staining of liver sections. Representative images showing apoptotic cells (green) and nuclei (blue) labeled by TUNEL and DAPI staining, respectively (scale bar = 1 mm). TUNEL-positive cells increased markedly in the MPs + BPA group compared to controls, indicating enhanced apoptosis after combined exposure

Exposure to MPs, BPA, or their combination triggered stress and apoptosis signaling in *M. salmoides*. *HSP70* expression was significantly upregulated across all exposure groups, peaking in the co-exposure group (Fig. 4a). Pro-apoptotic markers *Bax* and *Caspase-3* were elevated, while the anti-apoptotic gene *Bcl-2* was downregulated (Fig. 4b–d). Caspase-3 enzyme activity also increased significantly, showing maximal induction under combined exposure (Fig. 4e). TUNEL assays confirmed a clear increase of staining in apoptotic cells in liver sections, most notably in the MPs + BPA group (Fig. 4g). Concurrently, hepatic ATP levels declined sharply in all treated groups, indicating impaired energy metabolism and cellular homeostasis (Fig. 4f).

### Ultrastructural alterations of brain mitochondria

**Fig. 5:**
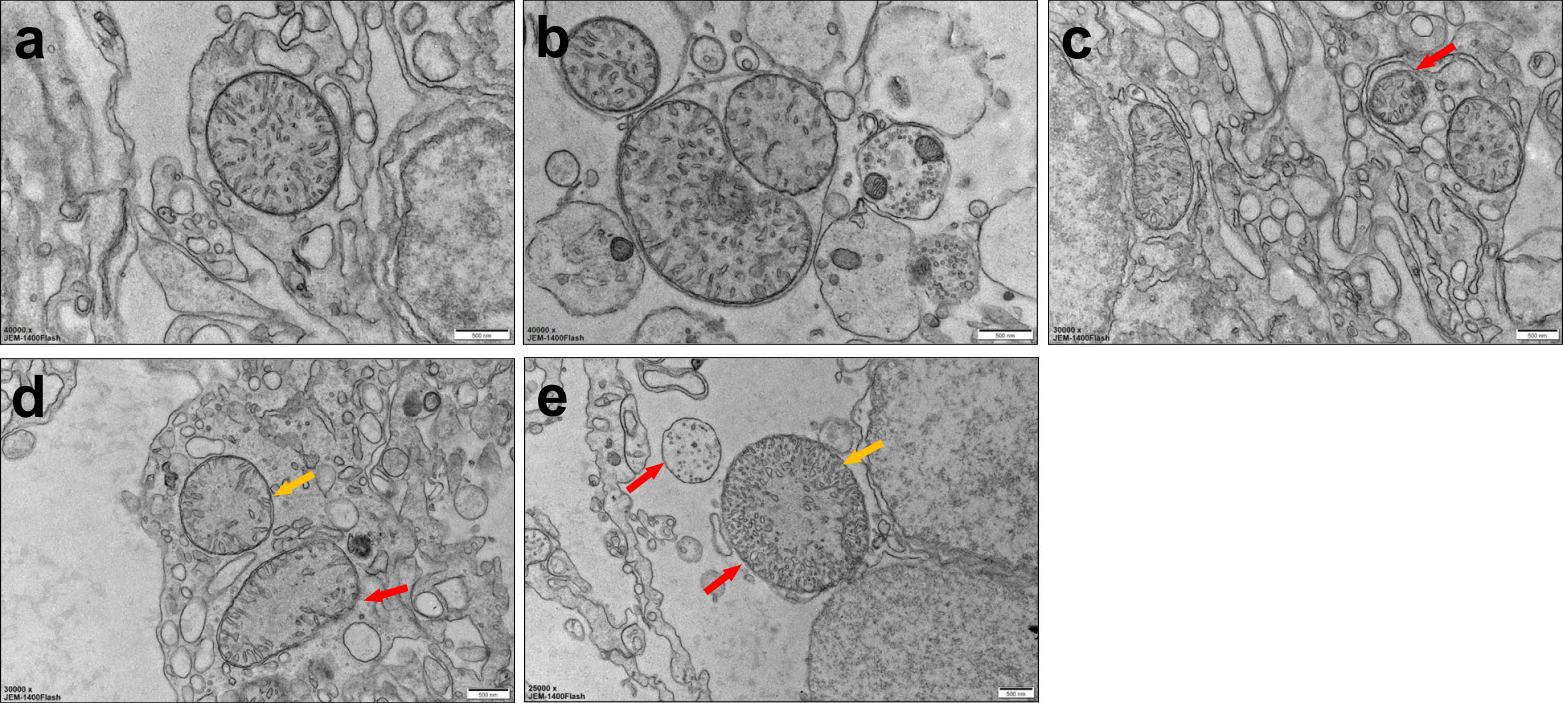
Mitochondrial ultrastructure in brain tissue of *M. salmoides* after exposure to MPs and BPA. TEM images of brain tissue from **a** control, **b** solvent control, **c** MPs, **d** BPA, and **e** MPs + BPA groups. Mitochondria in the co-exposure group **e** exhibited pronounced swelling, cristae disruption, and vacuolar degeneration. Mild alterations were seen in **c** and **d**, while **a** and **b** maintained intact mitochondrial morphology. Red arrows indicate swollen mitochondria; yellow arrows denote disorganized or fragmented cristae (scale bar = 500 nm)

Transmission electron microscopy revealed mitochondrial abnormalities in the brain tissue of fish co-exposed to MPs + BPA. Notably, swollen mitochondria with fragmented or disorganized cristae were frequently observed, often accompanied by vacuolar degeneration (Fig. 5e). In contrast, mitochondria in the control group exhibited intact membranes, dense matrices, and well-defined cristae (Fig. 5a–b). Mild structural alterations were also observed in the BPA-only and MPs-only groups (Fig. 5c–d), but these changes were significantly less severe than those in the co-exposure group. These ultrastructural changes indicate a progressive mitochondrial injury in neural tissues under pollutant exposure, highlighting the synergistic toxicity of MP-BPA mixtures.

### BPA Binding to NRF2 and perturbation of Redox-associated signaling

**Fig. 6:**
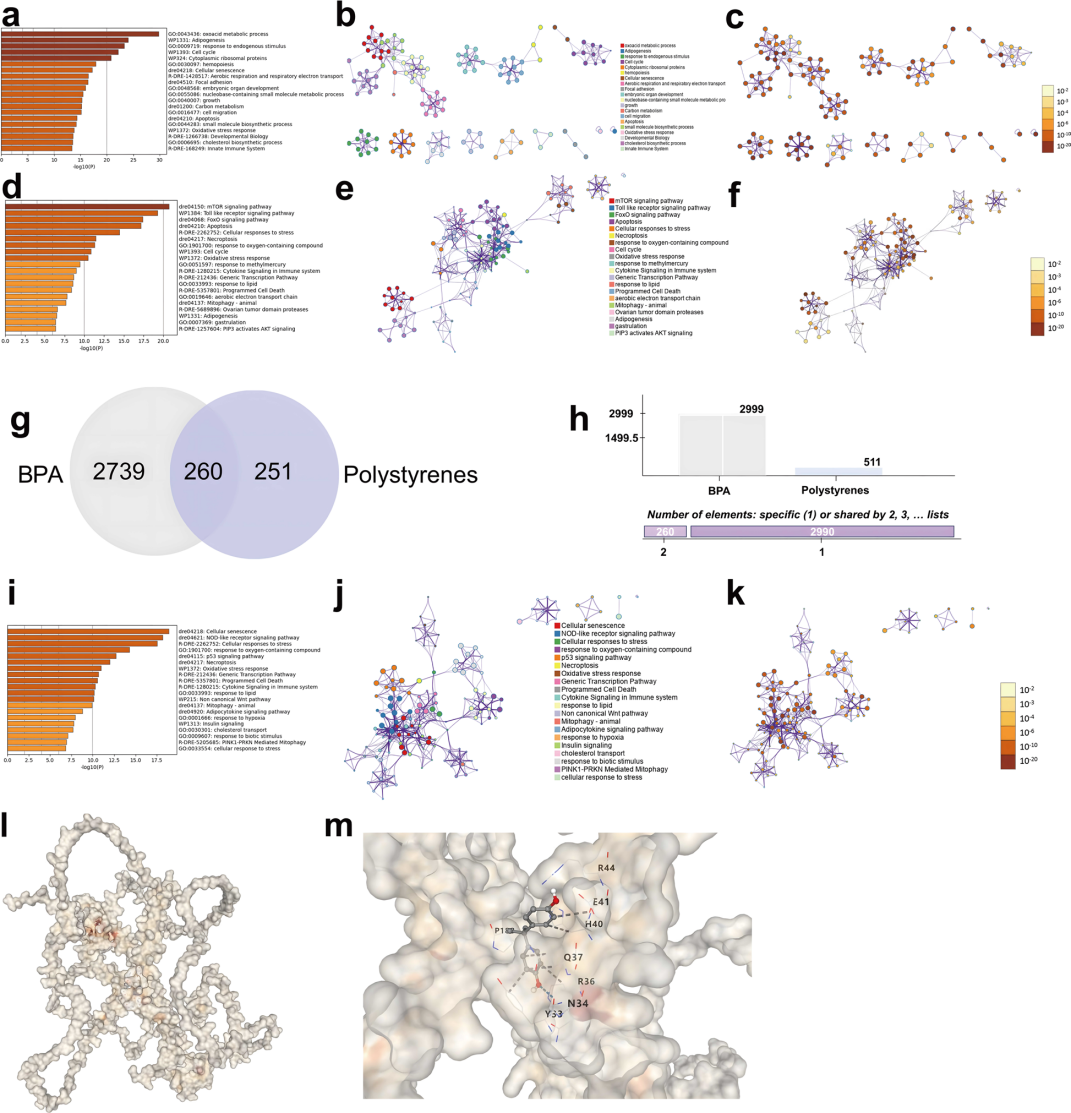
Bioinformatics and molecular docking analysis of BPA and polystyrene exposure. **a–c** GO, pathway enrichment, and network analysis of BPA targets. **d–f** Corresponding analyses for polystyrenes. **g–h** Venn diagram and element count showing 260 shared targets between BPA and polystyrenes. **i–k** Combined exposure enrichment analysis of overlapping targets. **l–m** Molecular docking of BPA with *M. salmoides* NRF2 protein showing stable binding at a hydrophobic cavity involving key residues (e.g., N34, Q37, Y33), indicating possible interference with antioxidant signaling. The original figure can be found in the supplementary materials

Molecular docking analyses demonstrated stable binding of BPA to the hydrophobic pocket of *M. salmoides* NRF2 protein, with a calculated affinity (binding energy) of –6.2 kcal/mol (Fig. 6l–m). Key residues mediating this interaction included Tyr33, Asn34, Gln37, and Arg36, formed hydrogen bonds and van der Waals forces, potentially distabilizing NRF2’s conformation and impairing its transcriptional activation of antioxidant genes.

In parallel, network toxicology analysis identified 260 overlapping gene targets between BPA and polystyrene exposure (Fig. 6g–h). GO enrichment of these shared targets revealed clusters associated with oxidative stress responses, lipid metabolism, and apoptotic regulation (Fig. 6a–c, i–k). Consistent with these findings, KEGG pathway analysis further highlighted significant enrichment in redox-related signaling cascades, including PI3K-Akt, mTOR, and FoxO pathways (Fig. 6d–f), suggesting convergent disruption of NRF2-mediated redox control as a plausible mechanism underlying MPs + BPA exposure compromise cellular antioxidant defense and promote hepatocellular injury.

### Increased susceptibility to NNV infection

**Fig. 7:**
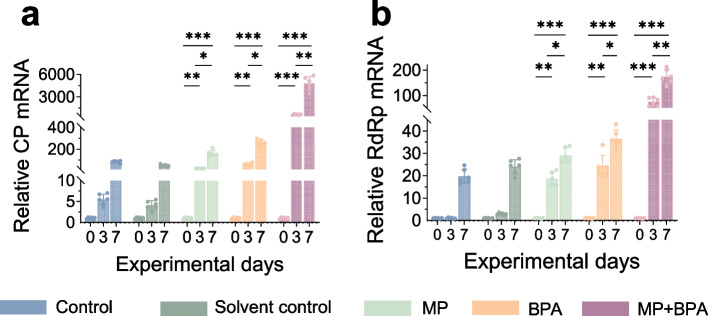
Brain expression of NNV CP and RdRp after MPs and BPA exposure. Relative mRNA levels of NNV *CP* and *RdRp* genes on days 0, 3, and 7 post-infection in different exposure groups

To assess the impact of prior exposure to MPs and BPA on host immune resistance, *M. salmoides* were subjected to NNV challenge following 14 days of treatment. Transcriptional analysis revealed significant upregulation of viral gene markers, including NNV capsid protein (*CP*) and RNA-dependent RNA polymerase (*RdRp*), in all exposed groups compared with the control (*P* < 0.05). Notably, the highest viral loads were detected in the MPs + BPA group, with peak viral genome observed at both 3 and 7 day post-infection (dpi) (Fig. 7).

## Discussion

### Summary of key findings

This study identifies a mechanistic cascade in which co-exposure to MPs and BPA impairs antiviral defense in *M. salmoides. This impairment is* mediated by oxidative stress, bioenergetic disruption, and apoptosis. At the core of this process is the suppression of NRF2 signaling, the master regulator of antioxidant defense, which integrates these effects into a unified toxicological pathway rather than treating them as isolated outcomes.

Compared with single exposures, co-exposure produced stronger inhibition of NRF2 and its downstream targets (SOD1, CAT, GPx), indicating enhanced sensitivity of this pathway to combined chemical stress (Geremia et al. 2023; Subaramaniyam et al. 2023). The observed upregulation of Keap1 suggests a feedback regulatory mechanism that restricts NRF2 nuclear translocation in response to sustained oxidative stress. While similar NRF2–Keap1 regulation has been reported in mammalian and teleost models (Feng et al. 2022a, b; Santos et al. 2021; Caballero-Gallardo et al. 2016), our results extend its relevance to freshwater carnivorous species of aquacultural importance.

**Fig. 8:**
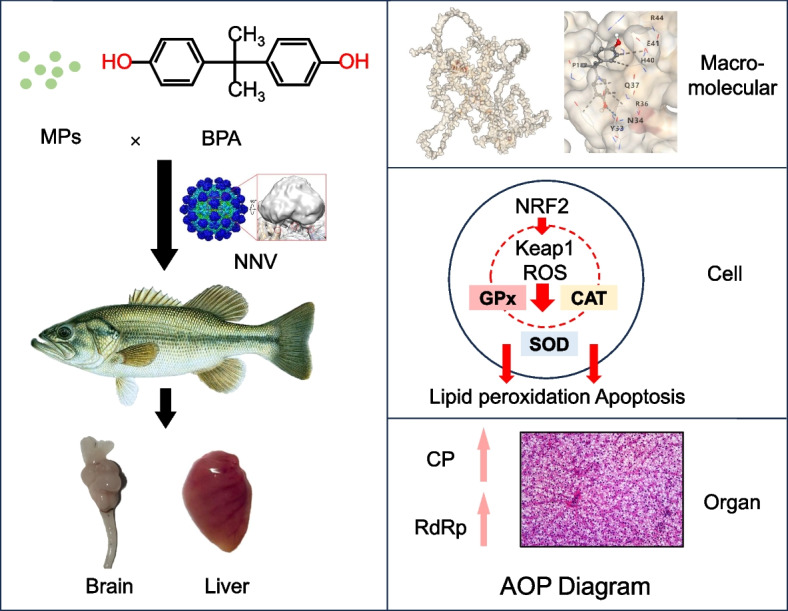
AOP of MPs and BPA induced hepatic toxicity in *M. salmoides*

Concurrently, mitochondrial dysfunction was evident, including ATP depletion and apoptotic activation, as indicated by an altered Bax/Bcl-2 ratio, elevated Caspase-3, and nuclear fragmentation, suggesting that energetic failure plays a key role in cell death (Santos et al. 2021). These findings align with rodent studies linking BPA-induced hepatic injury to oxidative stress and mitochondrial damage (Tang et al. 2024). Functionally, co-exposed fish exhibited significantly increased viral loads following NNV challenge, indicating that subcellular stress responses translate into heightened susceptibility to infection. Molecular docking further revealed a direct interaction between BPA and NRF2, supporting a chemical inhibition mechanism and reinforcing NRF2 suppression as a molecular initiating event (MIE) within an oxidative stress–based adverse outcome pathway (AOP) (Fig. 8).

### Disruption of antioxidant defense via NRF2 suppression

NRF2, a master regulator of redox balance and cellular defense against electrophilic stress, was significantly suppressed under MPs and BPA co-exposure. This indicates a critical vulnerability of the antioxidant system to mixed chemical stress. The concomitant upregulation of Keap1 suggests a feedback mechanism that retainsNRF2 in the cytoplasm for proteasomal degradation, consistent with previous observations in BPA-exposed catfish and loach (Zheng et al. 2023; Wang et al. 2022). The suppression of NRF2 at environmentally relevant concentrations indicates that pollutant mixtures can induce synergistic redox interference beyond the effects of individual compounds.

Compared to classical antioxidant enzymes, NRF2 exhibited greater transcriptional sensitivity to combined exposure, suggesting it acts not only as a downstream effector but also as an early and amplifiable signal target within the oxidative stress cascade. Similar patterns have been reported in murine hepatic models, where chronic BPA exposure led to persistent repression of NRF2 through oxidative and inflammatory signaling (Feng et al. 2022a, b). In aquatic species, MPs likely exacerbate cytotoxicity by enhancing the intracellular delivery or bioavailability of hydrophobic contaminants such as BPA (Li et al. 2021).

The observed ROS and MDA accumulation corroborates the disruption of the NRF2 pathway. Interestingly, the absence of MDA elevation in the BPA-only group suggests that MPs modulate the oxidative impact of BPA. This could result from MPs’ physical vector effects, increased cellular uptake, or altered intracellular fate of BPA, as shown in zebrafish embryos and larvae (Cimmino et al. 2020; Qin et al. 2020). Molecular docking further revealed stable binding between BPA and key residues in NRF2’s hydrophobic motif (e.g., Tyr33, Asn34), potentially disrupting protein folding or nuclear translocation. Similar ligand–receptor interference has been proposed for endocrine disruption mechanisms in grass carp hepatocytes (Chen et al. 2022), indicating a conserved mode of NRF2 inhibition among structurally diverse pollutants.

### Cellular energy deficiency and apoptotic response

Energy metabolic disruptions often accompany oxidative stress, particularly when mitochondrial function is compromised. In this study, a pronounced synergistic decline in hepatic ATP levels was observed exclusively under MP and BPA co-exposure, suggesting that pollutant mixtures disrupt bioenergetic homeostasis more severely than individual compounds. This interaction reveals and amplifies latent mitochondrial vulnerabilities exacerbated by chemical mixtures (Azevedo et al. 2019). While previous studies in zebrafish linked BPA to mitochondrial ATP synthesis impairment at higher concentrations or extended exposures (Pelaia et al. 2023), however, our findings indicate that MPs may potentiate BPA-induced toxicity by facilitating intracellular delivery or directly targeting mitochondrial structures (Xue et al. 2024), thereby lowering the effective threshold for toxicity.

Mitochondrial stress is a well-established initiator of apoptosis. In this study, ATP depletion was accompanied by distinct apoptotic responses, including an elevated Bax/Bcl-2 ratio, increased caspase-3 activation, and TUNEL-positive nuclei in the co-exposed groups. These features support a mitochondria-mediated pathway of programmed cell death, consistent with previous reports in fish liver following pollutant exposure (Xue et al. 2021; Wang et al. 2019; Zha et al. 2017). Notably, our use of juvenile fish and subchronic exposure differs from most studies focusing on embryos or early life stages (Raftery et al. 2017; Zhang et al. 2023), demonstrating that mature tissues remain highly susceptible to apoptotic stimulation under environmentally relevant pollutant combinations.

Although both ROS and MDA accumulation were evident in the co-exposure group, the concurrent ATP decline suggests that oxidative stress alone may not fully account for cell death. Instead, impaired mitochondrial bioenergetics appears to serve as a central link connecting redox imbalance to apoptosis. Similar coupling between energy failure and apoptosis has been documented in zebrafish and carp following exposure to plasticizers or endocrine disruptors (Hou et al. 2024; Raftery et al. 2017), reinforcing the hypothesis that mitochondria function as both primary targets and amplifiers of pollutant-induced toxicity.

### Impaired host defense under co-exposure

The increased replication of NNV observed under MPs + BPA co-exposure indicates that pollutant mixtures can compromise host antiviral defenses. While oxidative stress and apoptosis have each been independently linked to immune suppression, their convergence under combined exposure may synergistically weaken the host’s capacity to control viral infection. Elevated ROS levels can disrupt redox-sensitive immune signaling, whereas energy depletion and apoptotic activation impair the cellular capacity to limit viral spread (Zhang et al. 2023). This effect was not observed in the single-exposure groups, highlighting the important role of chemical interactions in exacerbating mixture toxicity. Possible explanations include surpassing the cellular tolerance threshold or MPs acting as vectors that enhance BPA uptake, thereby amplifying toxicity (Abdel-Latif et al. 2022).

Previous studies have mainly focused on pollutant-induced modulation of immune genes or direct viral inhibition. In contrast, our findings suggest a mechanistic pathway in which mitochondrial injury, redox imbalance, and programmed cell death collectively contribute to heightened viral susceptibility. TEM analysis revealed structural disruptions in brain mitochondria, including swelling, cristae fragmentation, and vacuolar degeneration, providing anatomical evidence of neural stress that may underlie the enhanced viral replication observed in the co-exposure group. These mitochondrial alterations have also been associated with neurotoxicity in other aquatic models (Sánchez-González et al. 2025). Given that NNV primarily targets neural tissues, mitochondrial damage within these regions may compromise local antiviral defenses, thereby facilitating viral propagation.

### Ecotoxicological implications

Most ecotoxicological evaluations have traditionally focused on individual chemicals, yet aquatic organisms are frequently exposed to complex pollutant mixtures in natural environments. The MP–BPA co-exposure examined here represents a realistic environmental scenario, particularly in freshwater aquaculture systems where plastic debris and chemical additives often coexist (Stumm et al., 1982). Our results demonstrate that such combinations can trigger biological outcomes—such as enhanced viral susceptibility and mitochondrial dysfunction—that are not predicted by single-compound effects, emphasizing the need to reorient current frameworks toward mixture-based toxicity assessments.

The selection of *M. salmoides*, a carnivorous fish of both ecological and commercial significance, enhances the relevance of our findings. Its high metabolic demand and immune sensitivity make it a suitable sentinel species for detecting redox-related immunotoxic endpoints. Incorporating mixture-responsive biomarkers, such as NRF2 inhibition, ATP depletion, and viral susceptibility, into environmental monitoring strategies could improve ecological risk prediction accuracy (Costantini et al. 2023).

Furthermore, this study supports the application of the adverse outcome pathway (AOP) framework for organizing mechanistic toxicity data. By integrating molecular (NRF2 inhibition), cellular (oxidative stress and apoptosis), and organismal (NNV susceptibility) responses, our work demonstrates the practical value of AOP-based reasoning in addressing complex mixture exposures. Future research should extend this approach across different species, developmental stages, and pollutant combinations to further validate redox-driven immunotoxic AOPs in freshwater ecosystems.

## Conclusion

This study demonstrates that co-exposure to MPs and BPA synergistically disrupts NRF2-mediated antioxidant defense, leading to oxidative stress, apoptosis, and increased viral susceptibility in *M. salmoides*. From a mechanistic standpoint, our findings identify NRF2 suppression as the key molecular link between pollutant mixtures and compromised immune capacity. Ecologically, the results highlight that chemical co-contaminants can impair fish health beyond conventional toxicological endpoints. From a management perspective, these findings emphasize the urgent need to incorporate mixture toxicity and immune-related biomarkers into freshwater ecological risk assessments and aquaculture sustainability frameworks.

## Supplementary Information

Supplementary Material 1.

## Data Availability

The data supporting the findings of this study are available from the authors upon reasonable request.
