# Publisher Correction: Co-exposure to microplastics and bisphenol A increases viral susceptibility in largemouth bass (*Micropterus salmoides*) via oxidative stress

**DOI:** 10.1007/s44307-025-00089-1

**Published:** 2025-12-11

**Authors:** Jie Gao, Junzhe Zhang, Rui Zheng, Jing Jiang, Siyou Huang, Qijin Miao, Bingya Wu, Wanting Tang, Jianguo He, Junfeng Xie

**Affiliations:** State Key Laboratory of Biocontrol, Southern Marine Science and Engineering Guangdong Laboratory (Zhuhai), China-ASEAN Belt and Road Joint Laboratory On Mariculture Technology, Guangdong Provincial Key Laboratory of Aquatic Economic Animals, School of Life Sciences, Sun Yat-Sen University, Guangzhou, 510,275 China

**Correction: Advanced Biotechnology 3, 31 (2025)**

**https://doi.org/10.1007/s44307-025–00085-5**

Following publication of the original article (Gao et al. 2025), it was reported that the Graphical Abstract needed to be removed.

The original article (Gao et al. 2025) has been corrected.
