# Supplementary material for: Co-exposure to microplastics and bisphenol A increases viral susceptibility in largemouth bass (Micropterus salmoides) via oxidative stress
Source: Adv Biotechnol (Singap). 2025 Nov 6;3(4):31. doi: 10.1007/s44307-025-00085-5 (PMC12592606; doi:10.1007/s44307-025-00085-5)
Supplement: Supplementary file 1 — Supplementary Material 1. [file 44307_2025_85_MOESM1_ESM.docx]

**Co-exposure to Microplastics and Bisphenol A Increases Viral Susceptibility in Largemouth Bass (*Micropterus salmoides*) via Oxidative Stress**

**Jie Gao^1^, Junzhe Zhang^1^, Rui Zheng, Jing Jiang,** **Siyou Huang, Qijin Miao, Bingya Wu, Wanting Tang,** **Jianguo He, Junfeng Xie**^*^

State Key Laboratory of Biocontrol, Southern Marine Science and Engineering Guangdong Laboratory (Zhuhai), China-ASEAN Belt and Road Joint Laboratory on Mariculture Technology, Guangdong Provincial Key Laboratory of Aquatic Economic Animals, School of Life Sciences, Sun Yat-sen University, China

1 These authors contributed equally to this work.

* Corresponding Author:

Junfeng Xie, Ph.D., Associate Professor

School of Life Sciences, Sun Yat-sen University

Guangzhou, 510275, China

Tel: +86 20 84113793; Fax: +86 20 84113229;

E-mail: [xiejf@mail.sysu.edu.cn](mailto:xiejf@mail.sysu.edu.cn)


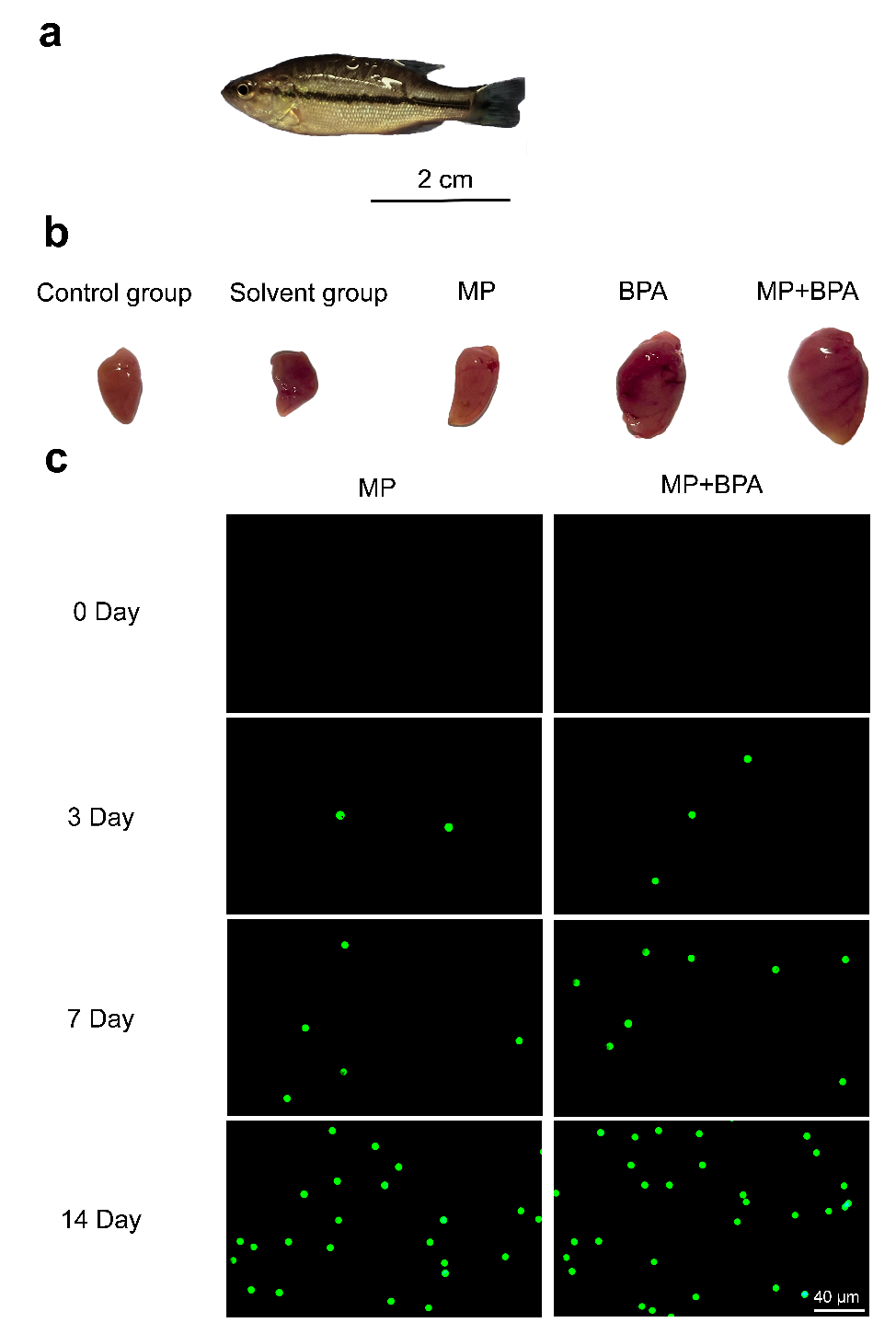


Fig. S1 Experimental fish, liver phenotype, and microplastic distribution. a: Image of the experimental animal (*M. salmoides*) used in the exposure study. b: Gross morphological differences in liver appearance between control and exposed fish at the end of the 14-day experiment. c: Fluorescent micrographs showing the distribution of microplastics (green) in liver tissue of MPs and MPs + BPA groups at 0, 3, 7, and 14 days. Accumulation of fluorescent particles was more pronounced in the MPs + BPA group over time

Table S1 Specific amplification primer used in this study

| Primer name | Primer sequence (5'to3') | Amplification target |
| --- | --- | --- |
| SOD-F | ACGGACAAGGTGCTCAGC | qRT-PCR |
| SOD-R | CCAGACGTCCACCAGCAT |  |
| CAT-F | TGACCTTCAAGCAGGCCG |  |
| CAT-R | GCTGCTCCACCTCTGCAA |  |
| GPx-F | TCCCCTTGCGATGATGCC |  |
| GPx-R | GGCTCCCCATCAGGACCA |  |
| NRF2-F | CACCAGGGATGGCAGTGG |  |
| NRF2-R | CCAGACAGCCACCCAGTG |  |
| HSP70-F | CCACACCCAGCTACGTGG |  |
| HSP70-R | TGCGGCCAATCAGTCGTT |  |
| KEAP1-F | ATCAGCCGCGATGAGCTC |  |
| KEAP1-R | GTTCTCCCGGTCGTAGCG |  |
| Bax-F | GTTCCTTGGAGGCACGCT |  |
| Bax-R | AGCCGCACATGATGAGCA |  |
| Bcl-F | TGTTGCGGATCAGGAGGC |  |
| Bcl-R | CCAATGCAGGCCTCCACA |  |
| Caspase-3-F | ACAGCCAACGGTTCCTGG |  |
| Caspase-3-R | CTTGCGGTTGACTCGGGT |  |
| EF-1α-F | TGCTGCTGGTGTTGGTGAGTT |  |
| EF-1α-R | TTCTGGCTGTAAGGGGGCTC |  |
